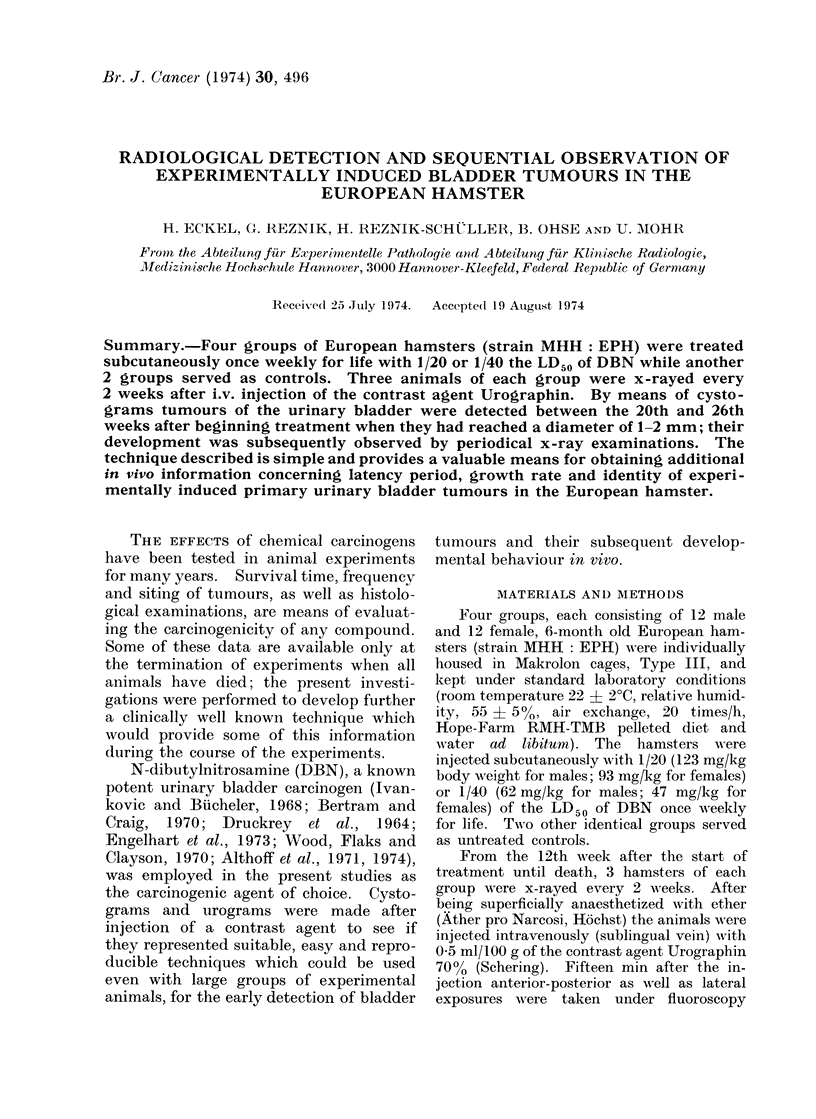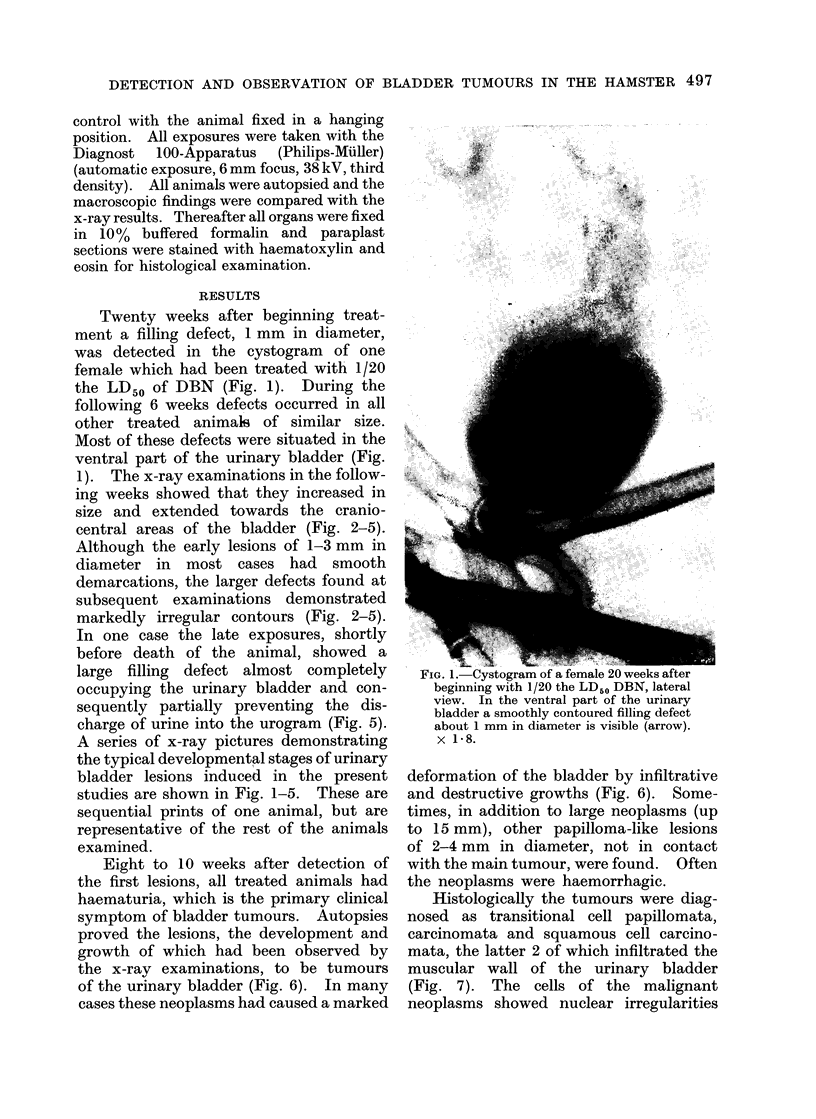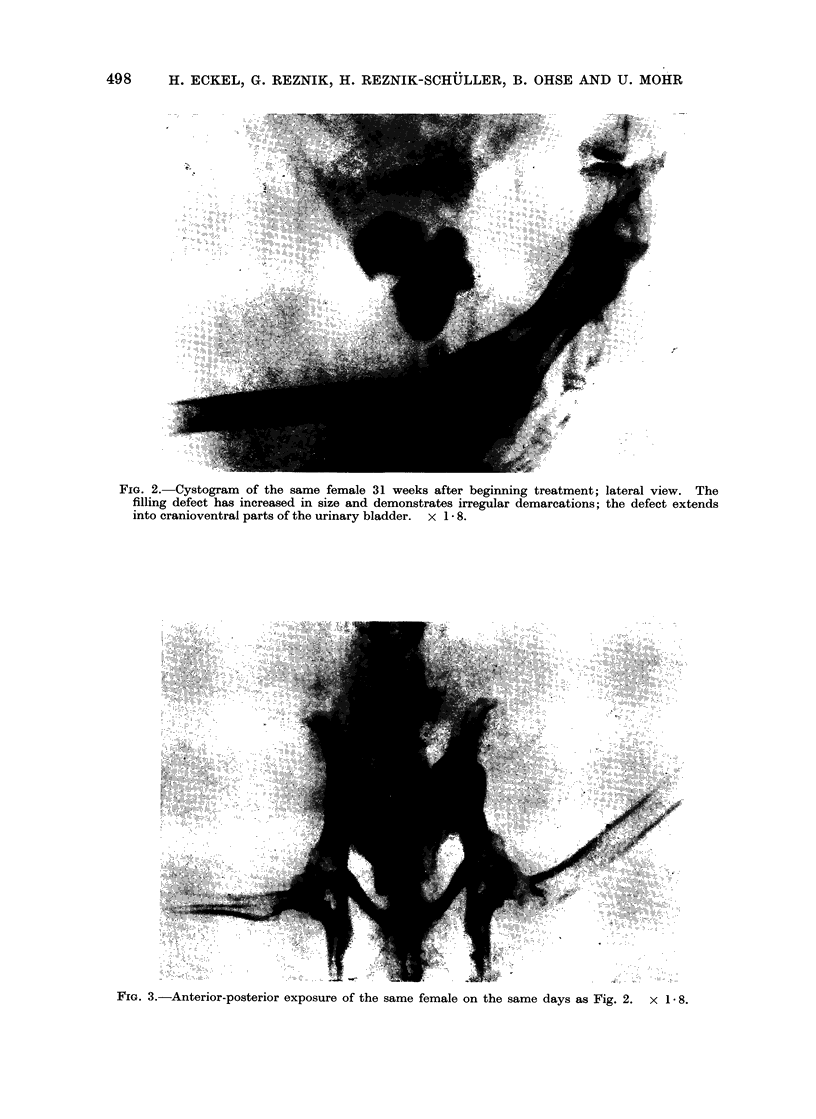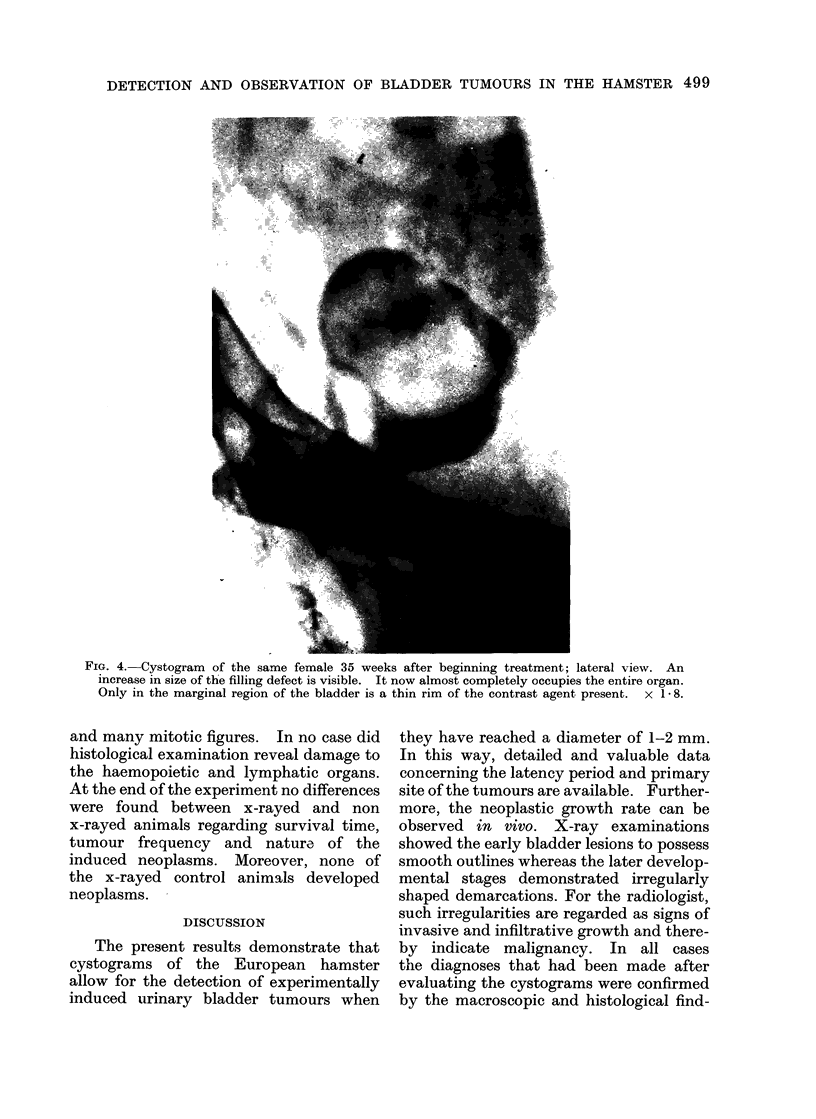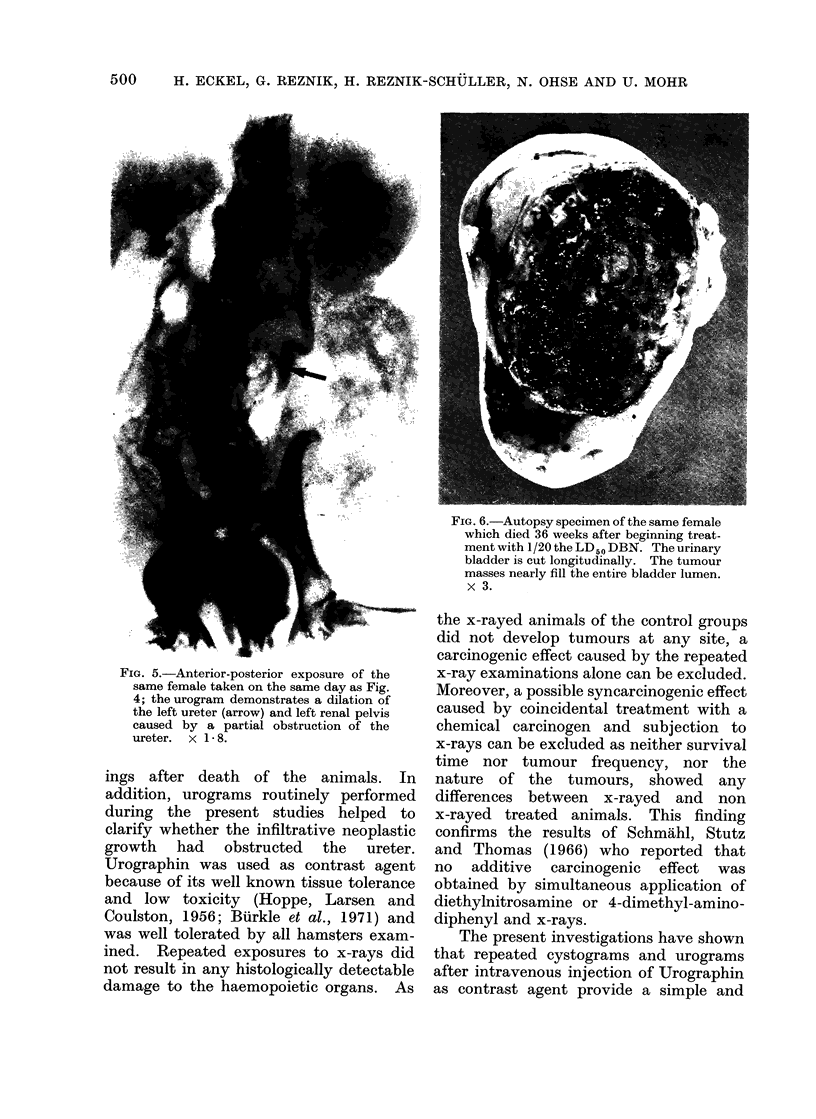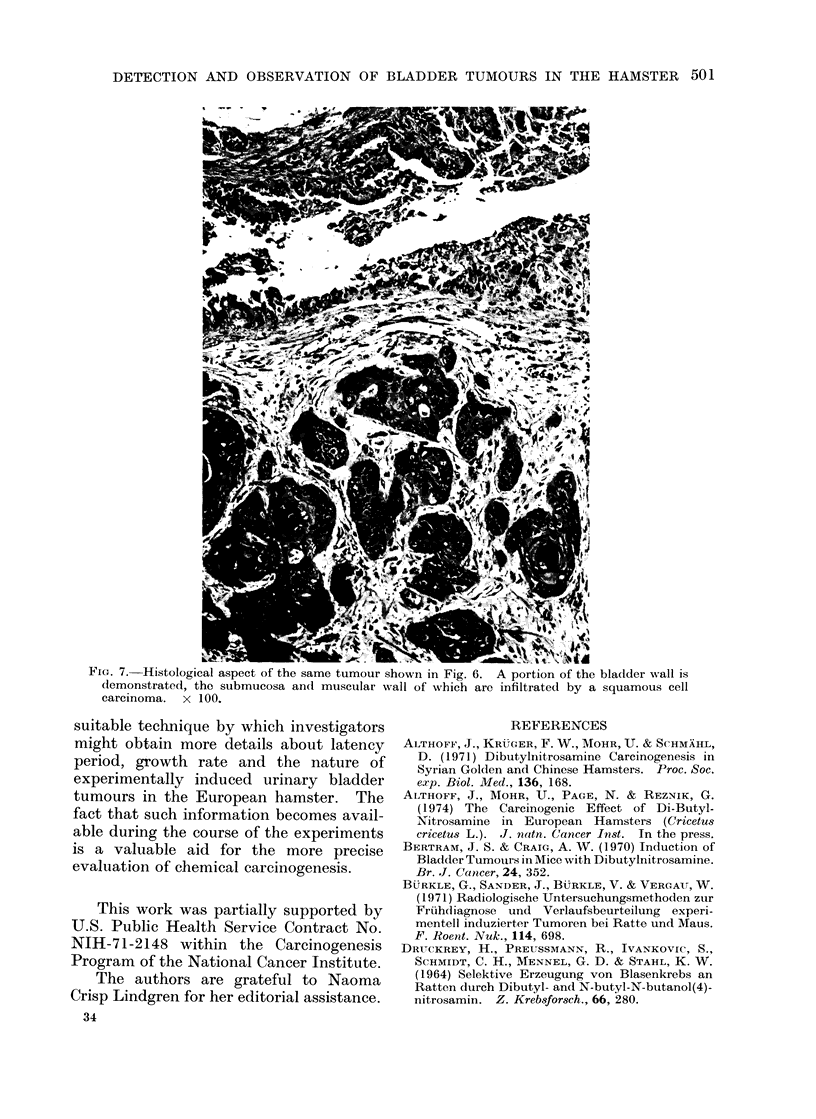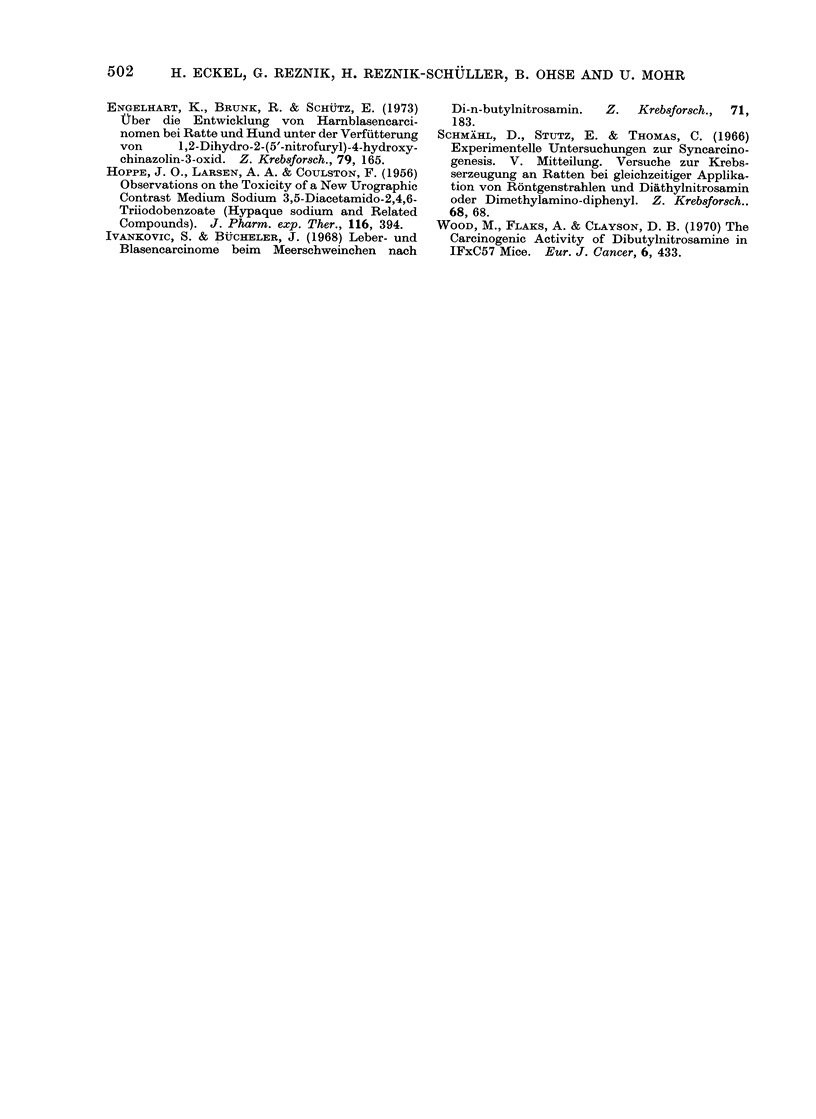# Radiological Detection and Sequential Observation of Experimentally Induced Bladder Tumours in the European Hamster

**DOI:** 10.1038/bjc.1974.228

**Published:** 1974-12

**Authors:** H. Eckel, G. Reznik, H. Reznik-Schüller, B. Ohse, U. Mohr

## Abstract

**Images:**


*Br. J. Cancer* (1974) **30**, 496

### RADIOLOGICAL DETECTION AND SEQUENTIAL OBSERVATION OF EXPERIMENTALLY INDUCED BLADDER TUMOURS IN THE EUROPEAN HAMSTER

H. ECKEL, G. REZNIK, H. REZNIK-SCHÜLLER, B. OHSE AND U. MOHR

*From the Abteilung für Experimentelle Pathologie and Abteilung für Klinische Radiologie, Medizinische Hochschule Hannover,* 3000 *Hannover-Kleefeld, Federal Republic of Germany*

Received 25 July 1974. Accepted 19 August 1974

**Summary.—Four groups of European hamsters (strain MHH : EPH) were treated subcutaneously once weekly for life with 1/20 or 1/40 the $LD_{50}$ of DBN while another 2 groups served as controls. Three animals of each group were x-rayed every 2 weeks after i.v. injection of the contrast agent Urographin. By means of cystograms tumours of the urinary bladder were detected between the 20th and 26th weeks after beginning treatment when they had reached a diameter of 1–2 mm; their development was subsequently observed by periodical x-ray examinations. The technique described is simple and provides a valuable means for obtaining additional *in vivo* information concerning latency period, growth rate and identity of experimentally induced primary urinary bladder tumours in the European hamster.**

THE EFFECTS of chemical carcinogens have been tested in animal experiments for many years. Survival time, frequency and siting of tumours, as well as histological examinations, are means of evaluating the carcinogenicity of any compound. Some of these data are available only at the termination of experiments when all animals have died; the present investigations were performed to develop further a clinically well known technique which would provide some of this information during the course of the experiments.

N-dibutylnitrosamine (DBN), a known potent urinary bladder carcinogen (Ivankovic and Bücheler, 1968; Bertram and Craig, 1970; Druckrey *et al.*, 1964; Engelhart *et al.*, 1973; Wood, Flaks and Clayson, 1970; Althoff *et al.*, 1971, 1974), was employed in the present studies as the carcinogenic agent of choice. Cystograms and urograms were made after injection of a contrast agent to see if they represented suitable, easy and reproducible techniques which could be used even with large groups of experimental animals, for the early detection of bladder tumours and their subsequent developmental behaviour *in vivo.*

### MATERIALS AND METHODS

Four groups, each consisting of 12 male and 12 female, 6-month old European hamsters (strain MHH : EPH) were individually housed in Makrolon cages, Type III, and kept under standard laboratory conditions (room temperature 22 ± 2°C, relative humidity, 55 ± 5%, air exchange, 20 times/h, Hope-Farm RMH-TMB pelleted diet and water *ad libitum*). The hamsters were injected subcutaneously with 1/20 (123 mg/kg body weight for males; 93 mg/kg for females) or 1/40 (62 mg/kg for males; 47 mg/kg for females) of the $LD_{50}$ of DBN once weekly for life. Two other identical groups served as untreated controls.

From the 12th week after the start of treatment until death, 3 hamsters of each group were x-rayed every 2 weeks. After being superficially anaesthetized with ether (Äther pro Narcosi, Höchst) the animals were injected intravenously (sublingual vein) with 0·5 ml/100 g of the contrast agent Urographin 70% (Schering). Fifteen min after the injection anterior-posterior as well as lateral exposures were taken under fluoroscopy control with the animal fixed in a hanging position. All exposures were taken with the Diagnost 100-Apparatus (Philips-Müller) (automatic exposure, 6 mm focus, 38 kV, third density). All animals were autopsied and the macroscopic findings were compared with the x-ray results. Thereafter all organs were fixed in 10% buffered formalin and paraplast sections were stained with haematoxylin and eosin for histological examination.

RESULTS

Twenty weeks after beginning treatment a filling defect, 1 mm in diameter, was detected in the cystogram of one female which had been treated with 1/20 the $LD_{50}$ of DBN (Fig. 1). During the following 6 weeks defects occurred in all other treated animals of similar size. Most of these defects were situated in the ventral part of the urinary bladder (Fig. 1). The x-ray examinations in the following weeks showed that they increased in size and extended towards the cranio-central areas of the bladder (Fig. 2–5). Although the early lesions of 1–3 mm in diameter in most cases had smooth demarcations, the larger defects found at subsequent examinations demonstrated markedly irregular contours (Fig. 2–5). In one case the late exposures, shortly before death of the animal, showed a large filling defect almost completely occupying the urinary bladder and consequently partially preventing the discharge of urine into the urogram (Fig. 5). A series of x-ray pictures demonstrating the typical developmental stages of urinary bladder lesions induced in the present studies are shown in Fig. 1–5. These are sequential prints of one animal, but are representative of the rest of the animals examined.

Eight to 10 weeks after detection of the first lesions, all treated animals had haematuria, which is the primary clinical symptom of bladder tumours. Autopsies proved the lesions, the development and growth of which had been observed by the x-ray examinations, to be tumours of the urinary bladder (Fig. 6). In many cases these neoplasms had caused a marked

FIG. 1.—Cystogram of a female 20 weeks after beginning with 1/20 the $LD_{50}$ DBN, lateral view. In the ventral part of the urinary bladder a smoothly contoured filling defect about 1 mm in diameter is visible (arrow). × 1·8.

deformation of the bladder by infiltrative and destructive growths (Fig. 6). Sometimes, in addition to large neoplasms (up to 15 mm), other papilloma-like lesions of 2–4 mm in diameter, not in contact with the main tumour, were found. Often the neoplasms were haemorrhagic.

Histologically the tumours were diagnosed as transitional cell papillomata, carcinomata and squamous cell carcinomata, the latter 2 of which infiltrated the muscular wall of the urinary bladder (Fig. 7). The cells of the malignant neoplasms showed nuclear irregularities

FIG. 2.—Cystogram of the same female 31 weeks after beginning treatment; lateral view. The filling defect has increased in size and demonstrates irregular demarcations; the defect extends into cranioventral parts of the urinary bladder. × 1·8.

FIG. 3.—Anterior-posterior exposure of the same female on the same days as Fig. 2. × 1·8.

FIG. 4.—Cystogram of the same female 35 weeks after beginning treatment; lateral view. An increase in size of the filling defect is visible. It now almost completely occupies the entire organ. Only in the marginal region of the bladder is a thin rim of the contrast agent present. × 1·8.

and many mitotic figures. In no case did histological examination reveal damage to the haemopoietic and lymphatic organs. At the end of the experiment no differences were found between x-rayed and non x-rayed animals regarding survival time, tumour frequency and nature of the induced neoplasms. Moreover, none of the x-rayed control animals developed neoplasms.

### DISCUSSION

The present results demonstrate that cystograms of the European hamster allow for the detection of experimentally induced urinary bladder tumours when they have reached a diameter of 1–2 mm. In this way, detailed and valuable data concerning the latency period and primary site of the tumours are available. Furthermore, the neoplastic growth rate can be observed *in vivo*. X-ray examinations showed the early bladder lesions to possess smooth outlines whereas the later developmental stages demonstrated irregularly shaped demarcations. For the radiologist, such irregularities are regarded as signs of invasive and infiltrative growth and thereby indicate malignancy. In all cases the diagnoses that had been made after evaluating the cystograms were confirmed by the macroscopic and histological find-

FIG. 5.—Anterior-posterior exposure of the same female taken on the same day as Fig. 4; the urogram demonstrates a dilation of the left ureter (arrow) and left renal pelvis caused by a partial obstruction of the ureter. × 1·8.

FIG. 6.—Autopsy specimen of the same female which died 36 weeks after beginning treatment with 1/20 the $LD_{50}$ DBN. The urinary bladder is cut longitudinally. The tumour masses nearly fill the entire bladder lumen. × 3.

ings after death of the animals. In addition, urograms routinely performed during the present studies helped to clarify whether the infiltrative neoplastic growth had obstructed the ureter. Urographin was used as contrast agent because of its well known tissue tolerance and low toxicity (Hoppe, Larsen and Coulston, 1956; Bürkle *et al.*, 1971) and was well tolerated by all hamsters examined. Repeated exposures to x-rays did not result in any histologically detectable damage to the haemopoietic organs. As the x-rayed animals of the control groups did not develop tumours at any site, a carcinogenic effect caused by the repeated x-ray examinations alone can be excluded. Moreover, a possible syncarcinogenic effect caused by coincidental treatment with a chemical carcinogen and subjection to x-rays can be excluded as neither survival time nor tumour frequency, nor the nature of the tumours, showed any differences between x-rayed and non x-rayed treated animals. This finding confirms the results of Schmähl, Stutz and Thomas (1966) who reported that no additive carcinogenic effect was obtained by simultaneous application of diethylnitrosamine or 4-dimethyl-amino-diphenyl and x-rays.

The present investigations have shown that repeated cystograms and urograms after intravenous injection of Urographin as contrast agent provide a simple and

FIG. 7.—Histological aspect of the same tumour shown in Fig. 6. A portion of the bladder wall is demonstrated, the submucosa and muscular wall of which are infiltrated by a squamous cell carcinoma. × 100.

suitable technique by which investigators might obtain more details about latency period, growth rate and the nature of experimentally induced urinary bladder tumours in the European hamster. The fact that such information becomes available during the course of the experiments is a valuable aid for the more precise evaluation of chemical carcinogenesis.

This work was partially supported by U.S. Public Health Service Contract No. NIH-71-2148 within the Carcinogenesis Program of the National Cancer Institute.

The authors are grateful to Naoma Crisp Lindgren for her editorial assistance.

REFERENCES

ALTHOFF, J., KRÜGER, F. W., MOHR, U. & SCHMÄHL, D. (1971) Dibutylnitrosamine Carcinogenesis in Syrian Golden and Chinese Hamsters. *Proc. Soc. exp. Biol. Med.*, **136**, 168.

ALTHOFF, J., MOHR, U., PAGE, N. & REZNIK, G. (1974) The Carcinogenic Effect of Di-Butyl-Nitrosamine in European Hamsters (*Cricetus cricetus* L.). *J. natn. Cancer Inst.* In the press.

BERTRAM, J. S. & CRAIG, A. W. (1970) Induction of Bladder Tumours in Mice with Dibutylnitrosamine. *Br. J. Cancer*, **24**, 352.

BÜRKLE, G., SANDER, J., BÜRKLE, V. & VERGAU, W. (1971) Radiologische Untersuchungsmethoden zur Frühdiagnose und Verlaufsbeurteilung experimentell induzierter Tumoren bei Ratte und Maus. *F. Roent. Nuk.*, **114**, 698.

DRUCKREY, H., PREUSSMANN, R., IVANKOVIC, S., SCHMIDT, C. H., MENNEL, G. D. & STAHL, K. W. (1964) Selektive Erzeugung von Blasenkrebs an Ratten durch Dibutyl- and N-butyl-N-butanol(4)-nitrosamin. *Z. Krebsforsch.*, **66**, 280.

ENGELHART, K., BRUNK, R. & SCHÜTZ, E. (1973) Über die Entwicklung von Harnblasencarcinomen bei Ratte und Hund unter der Verfütterung von 1,2-Dihydro-2-(5′-nitrofuryl)-4-hydroxychinazolin-3-oxid. *Z. Krebsforsch.*, **79**, 165.

HOPPE, J. O., LARSEN, A. A. & COULSTON, F. (1956) Observations on the Toxicity of a New Urographic Contrast Medium Sodium 3,5-Diacetamido-2,4,6-Triiodobenzoate (Hypaque sodium and Related Compounds). *J. Pharm. exp. Ther.*, **116**, 394.

IVANKOVIC, S. & BÜCHELER, J. (1968) Leber- und Blasencarcinome beim Meerschweinchen nach Di-n-butylnitrosamin. *Z. Krebsforsch.*, **71**, 183.

SCHMÄHL, D., STUTZ, E. & THOMAS, C. (1966) Experimentelle Untersuchungen zur Syncarcinogenesis. V. Mitteilung. Versuche zur Krebserzeugung an Ratten bei gleichzeitiger Applikation von Röntgenstrahlen und Diäthylnitrosamin oder Dimethylamino-diphenyl. *Z. Krebsforsch.*. **68**, 68.

WOOD, M., FLAKS, A. & CLAYSON, D. B. (1970) The Carcinogenic Activity of Dibutylnitrosamine in IFxC57 Mice. *Eur. J. Cancer*, **6**, 433.